# A new world for *Open Biology*

**DOI:** 10.1098/rsob.170002

**Published:** 2017-01-25

**Authors:** David M. Glover

The New Year is an excellent time to welcome the 38 new members to the editorial board of *Open Biology* (http://rsob.royalsocietypublishing.org/editorial-board). They are leading scientists from the USA, Canada and China who we hope will be champions for the journal in these countries and beyond. We are truly delighted to have these scientists join us.

It is also timely to evaluate our progress in establishing *Open Biology* as an open access journal edited by practising scientists to ensure fair and rapid review without the need for unnecessary rounds of revision. Our success is measured by the growing numbers of submissions and the quality of the papers we publish. The first is an easy metric, the second rather more difficult to assess. Most would agree that assessment of quality through the commonly used impact factors has been a contributory factor to the groundswell of dissatisfaction towards publishing felt by many biologists. This largely reflects the disproportionate influence of impact factors upon funding and careers. We accept that impact factors are most probably here to stay, but we do try and provide as many alternative means as we can for assessing publications (http://rsob.royalsocietypublishing.org/citation-metrics).

Open access was a first step towards making scientific findings quickly and widely accessible. A second step suggested by a growing populist movement in biology has been to use preprint servers. Indeed, the Society has long embraced the use of preprint servers and we encourage their use in biology in the hope that they will provide similar openness of communication as they have for many years to our colleagues in the physical sciences (https://royalsociety.org/journals/ethics-policies/media-embargo/). It remains to be seen how these will be embraced by biologists and indeed it could be argued that with fair review and rapid publication, they would be redundant.

Academies such as the Royal Society, the National Academy of the USA and EMBO continue to play an important role in publishing together with charitable bodies, such as the Company of Biologists and the many scientific societies who run their own journals. Together these provide an existing route to a fair publishing system that involves practising scientists. They continue a tradition established now over 350 years ago at a time when the Royal Society published its first journal. Then, as now, science was an international endeavour and a time when the Royal Society provided Mr van Leeuwenhoek the means of publishing his 190 or so letters. This necessitated that Henry Oldenberg learn Dutch to be able to translate the works into Latin or English. We still aspire to provide such a high level of editorial service!

We now live in a very different world, even to the one of a few decades ago, but we hope to go against the emerging trend for increased political isolationism and we continue to welcome authors to a truly international forum for scientific exchange. Let us all wish for a highly productive 2017 that meets our needs for free and open cooperation in science.

